# Protection from Amyloid *β* Peptide–Induced Memory, Biochemical, and Morphological Deficits by a Phosphodiesterase-4D Allosteric Inhibitor[Fn FN2]

**DOI:** 10.1124/jpet.119.259986

**Published:** 2019-11

**Authors:** Su-Ying Cui, Ming-Xin Yang, Yong-He Zhang, Victor Zheng, Han-Ting Zhang, Mark E. Gurney, Ying Xu, James M. O’Donnell

**Affiliations:** Department of Pharmacology, School of Basic Medical Science, Peking University, Beijing, China (S.-Y.C., Y.-H.Z.); Department of Pharmaceutical Sciences, School of Pharmacy and Pharmaceutical Sciences, State University of New York at Buffalo, Buffalo, New York (S.-Y.C., M.-X.Y., V.Z., Y.X., J.M.O.); Departments of Behavioral Medicine and Psychiatry, Physiology and Pharmacology, and Neuroscience, The Rockefeller Neurosciences Institute, West Virginia University Health Sciences Center, Morgantown, West Virginia (H.-T.Z.); and Tetra Discovery Partners Inc., Grand Rapids, Michigan (M.E.G.)

## Abstract

Recent imaging studies of amyloid and tau in cognitively normal elderly subjects imply that Alzheimer’s pathology can be tolerated by the brain to some extent due to compensatory mechanisms operating at the cellular and synaptic levels. The present study investigated the effects of an allosteric inhibitor of phosphodiesterase-4D (PDE4D), known as BPN14770 (2-(4-((2-(3-Chlorophenyl)-6-(trifluoromethyl)pyridin-4-yl)methyl)phenyl)acetic Acid), on impairment of memory, dendritic structure, and synaptic proteins induced by bilateral microinjection of oligomeric amyloid beta (A*β*_1–42_ into the hippocampus of humanized PDE4D (hPDE4D) mice. The hPDE4D mice provide a unique and powerful genetic tool for assessing PDE4D target engagement. Behavioral studies showed that treatment with BPN14770 significantly improved memory acquisition and retrieval in the Morris water maze test and the percentage of alternations in the Y-maze test in the model of A*β* impairment. Microinjection of oligomeric A*β*_1–42_ caused decreases in the number of dendrites, dendritic length, and spine density of pyramid neurons in the hippocampus. These changes were prevented by BPN14770 in a dose-dependent manner. Furthermore, molecular studies showed that BPN14770 prevented A*β*-induced decreases in synaptophysin, postsynaptic density protein 95, phosphorylated cAMP-response element binding protein (CREB)/CREB, brain-derived neurotrophic factor, and nerve growth factor inducible protein levels in the hippocampus. The protective effects of BPN14770 against A*β*-induced memory deficits, synaptic damage, and the alteration in the cAMP-meditated cell signaling cascade were blocked by H-89 (*N*-[2-(p-Bromocinnamylamino)ethyl]-5-isoquinolinesulfonamide dihydrochloride), an inhibitor of protein kinase A. These results suggest that BPN14770 may activate compensatory mechanisms that support synaptic health even with the onset of amyloid pathology in Alzheimer’s disease.

**SIGNIFICANCE STATEMENT:**

This study demonstrates that a phosphodiesterase-4D allosteric inhibitor, BPN14770, protects against memory loss and neuronal atrophy induced by oligomeric A*β*_1–42_. The study provides useful insight into the potential role of compensatory mechanisms in Alzheimer’s disease in a model of oligomeric A*β*_1–42_ neurotoxicity.

## Introduction

Human brain imaging studies with amyloid and tau reveal that Alzheimer’s pathology may develop without clinical dementia in 25%–35% of healthy 80-year-old subjects (Jack et al., 2017). These recent findings support and extend previous postmortem studies (Gosche et al., 2002; Mortimer et al., 2005; Boros et al., 2017, 2019). In the Religious Orders study, for example, up to 25% of subjects with pathologically significant Braak staging of I–IV were without clinical dementia at death (Mortimer et al., 2005). Why some individuals retain cognitive function despite the development of Alzheimer’s pathology while others do not is unclear.

Despite the lack of success with amyloid-directed therapies in late-stage human clinical trials (Karran and De Strooper, 2016), the amyloid and tau positron emission tomography imaging data imply that Alzheimer’s pathology can be tolerated by the brain to some extent due to compensatory mechanisms operating at the cellular and synaptic levels (Perneczky et al., 2019). For example, excitatory synapses in the cortex and hippocampus occur on dendritic spines. A small study by Boros et al. (2017) demonstrated that subjects without clinical dementia who nonetheless had developed Alzheimer’s pathology (Braak stages I–IV) had maintained dendritic spine density at similar levels to healthy, age-matched subjects. In contrast, spine density was decreased in subjects with Alzheimer’s disease. This suggests that compensatory mechanisms that maintain synaptic health may contribute to cognitive reserve even with the onslaught of Alzheimer’s pathology.

Nonmedical risk factors for the onset of clinical dementia in Alzheimer’s disease include low educational attainment, deafness, social isolation, and depression (Roe et al., 2007; Perneczky et al., 2019). Association of low educational attainment with risk for onset of dementia—or vice versa association of high educational attainment with reduced risk for dementia—has been reported across multiple studies of Alzheimer’s disease epidemiology. Educational attainment captures cognitive ability but more broadly reflects personality, motivation, and perseverance. Multiple large-scale, genome-wide association studies have associated genetic variation in the gene encoding phosphodiesterase-4D (PDE4D) with biologic variation in educational attainment (Lam et al., 2017; Xu et al., 2017; Lee et al., 2018; Savage et al., 2018; Davies et al., 2019; Gurney, 2019). PDE4D is a key modulator of cAMP signaling within dendritic spines and thereby synaptic processes underlying short- and long-term forms of memory (Baumgärtel et al., 2018). PDE4D inhibitors have also been shown to improve memory function without causing significant emetic-like behavior in rodents (Bruno et al., 2011; Zhang et al., 2017). The large size of the PDE4D gene, and its complex pattern of promoter utilization and alternative splicing associates genetic variation in dimeric forms of the PDE4D enzyme with biologic variation in human cognitive function (Bender and Beavo, 2006; Gurney, 2019). The importance of dimeric forms of PDE4D for normal brain function is underscored by the discovery of PDE4D missense mutations in an ultrarare, neurodevelopmental disorder known as acrodysostosis type 2 with or without hormone resistance (Lee et al., 2012; Linglart et al., 2012; Michot et al., 2012; Lynch et al., 2013).

The importance of phosphodiesterase-4 (PDE4) in processes regulating dendritic spine plasticity has been studied in model organisms. Dunce, the first mutation shown to affect associative memory in the *Drosophila* fruit fly was later found to be a deletion of the single PDE4 gene in the fly genome (Byers et al., 1981). The dunce deletion destroys the spatial and temporal patterning of cAMP signaling in the fly neurons important for olfactory learning (Gervasi et al., 2010). Studies in mice have placed PDE4D downstream from the calcium influx through the *N*-methyl-D-aspartate receptor in a pathway that leads to activation of calcium/calmodulin adenylate cyclase and phosphorylation of the cAMP-response element binding protein (CREB) transcription factor (Bailey et al., 1996; Kandel, 2009; Zhang et al., 2018). PDE4D is expressed in layer II/III cortical pyramidal neurons (Cherry and Davis, 1999), which play an important role in major mental disorders including fragile X syndrome, autism, and depression (Sinha et al., 2019; Zamarbide et al., 2019; Zhu et al., 2019). Furthermore, pharmacological inhibition or genetic knockdown of PDE4D activity promotes spine maturation in healthy adult mice and in a genetic model of fragile X syndrome (Gurney et al., 2017; Baumgärtel et al., 2018).

The role of PDE4 in modulating pathways important for Alzheimer’s pathogenesis has been studied extensively in mouse models using rolipram, a prototypical PDE4 allosteric inhibitor. The mammalian genome encodes four PDE4 enzymes, A–D, all of which are inhibited equally by rolipram (Bender and Beavo, 2006; Burgin et al., 2010). Rolipram is protective in models of in vitro amyloid beta (A*β*) neurotoxicity, prevents disease progression in amyloid protein precursor transgenic mice, and preserves synaptic connections (Vitolo et al., 2002; Gong et al., 2004; Shrestha et al., 2006; Smith et al., 2009). Although rolipram has been explored in patients with major depression, the compound is highly emetic, which prevents dosing in humans within the desired therapeutic range (Hebenstreit et al., 1989; Fleischhacker et al., 1992). Subtype-selective PDE4D allosteric inhibitors, in contrast to rolipram, achieve selectivity for PDE4D due to a single amino acid difference in the allosteric binding site on a regulatory domain known as upstream conserved region 2 (Burgin et al., 2010; Gurney et al., 2019). The key selectivity residue is a phenylalanine in PDE4D and a tyrosine in PDE4 subtypes A–C. This amino acid sequence difference is unique to PDE4D in humans and other primates. In nonprimates, including mice, rats, dogs, and other species, the key residue is a tyrosine as in the other PDE4 subtypes; therefore, there is no amino acid difference on upstream conserved region 2 among the different PDE4 subtypes that can be exploited for selectivity. Thus, PDE4D allosteric inhibitor pharmacology has not been studied previously in rodent models of Alzheimer’s disease.

To explore PDE4D pharmacology in mice, we humanized the mouse *PDE4D* gene by knockin of a single codon mutation of tyrosine 271 to phenylalanine in C57Bl6 mouse embryonic stem cells (Zhang et al., 2018). Humanized PDE4D (hPDE4D) mice were found to express a de novo, high-affinity binding site for BPN14770 (2-(4-((2-(3-Chlorophenyl)-6-(trifluoromethyl)pyridin-4-yl)methyl)phenyl)acetic Acid) with values of *K*_i_ = 2.8 + 1.1 nM, an increase in potency of over 40-fold. Therefore, we are able to treat hPDE4D mice with oral doses of BPN14770 (0.01–0.03 mg/kg) that are too low to engage other PDE4 subtypes in the brain (Zhang et al., 2018). Correspondingly, BPN14770 showed increased potency in humanized compared with wild-type C57Bl6 mice across multiple biomarkers and behavioral readouts of cAMP signaling, including an increase in brain cAMP, an increase in phosphorylation of CREB, augmentation of the late phase of hippocampal long-term potentiation, behavioral improvement in short- and long-term memory, and increased production of brain-derived neurotrophic factor (BDNF). Therefore, we sought to evaluate BPN14770 in an acute model of A*β* neurotoxicity based on microinjection of A*β*_1–42_ oligomers bilaterally into the hippocampus. This mimics aspects of Alzheimer’s pathology (Jiang et al., 2008; Cheng et al., 2010; Carrero et al., 2012; Wang et al., 2012, 2016) while allowing us to explore PDE4D pharmacology in hPDE4D mice.

## Materials and Methods

### 

#### Animals.

Humanized PDE4D transgenic mice were generated by inGenious Targeting Laboratory (Ronkonkoma, NY) and maintained as described previously (Zhang et al., 2018). A dimeric form of PDE4D, such as PDE4D7, is >99% identical between mouse and human across the 748 amino acid length of the protein. To humanize the mouse *PDE4D* gene by mutating tyrosine 271 to phenylalanine, a single point mutation of AC → TT was introduced into exon 9 of the mouse *PDE4D* gene by homologous recombination in C57Bl6 embryonic stem cells. The linearized vector contained a long homology arm extending ∼5.5 kb 5′ to the site of the AC → TT mutation in exon 9 and a short homology arm extending about ∼2.0 to a flippase recognition target–flanked neomycin cassette. Embryonic stem cell clones incorporating the AC → TT mutation were identified by polymerase chain reaction, implanted into surrogate females, and then chimeric mice with germ line transmission were identified and bred to homozygosity for the *hPDE4D* gene. All behavioral tests were carried out between 8:30 AM and 4:30 PM in a quiet room according to the National Institutes of Health Guide for the Care and Use of Laboratory Animals (revised in 2011, https://www.ncbi.nlm.nih.gov/books/NBK54050/). All procedures were approved by the Institutional Animal Care and Use Committee of the State University of New York at Buffalo.

#### Surgery.

Mice were anesthetized with ketamine and xylazine (100 and 10 mg/kg, i.p, respectively) and then placed in a stereotaxic apparatus. Two holes were drilled on the surface of the skull and guide cannulas (26 gauge; Plastic One) were implanted into the CA1 region of the hippocampus (anterior-posterior: −1.7 mm from bregma, medial-lateral: ± 0.8 mm from midline, and dorsal-ventral: −2.0 mm from dura) (Paxinos and Franklin, 2004; Wang et al., 2017). Dental cement and anchor screws were used to fix the cannula in place for microinjection. The mice were allowed to recover for 1 week before receiving any treatment. The location of the cannula/injection is shown in Fig. 3.

#### Drugs and Treatment.

A*β*_1–42_ (rPeptide) was dissolved in artificial cerebrospinal fluid (ACF) to a final concentration of 0.4 mg/ml, and before use was incubated at 37°C for 4 days to form aggregates. Aggregated A*β*_1–42_ (0.4 *μ*g in 1 *μ*l/side; rPeptide) or ACF was microinjected bilaterally into the CA1 of the hippocampus through an injection cannula in a volume of 1 *μ*l/side over a 5-minute period. BPN14770 was synthesized and prepared as previously described (Gurney et al., 2019). The protein kinase A (PKA) inhibitor *N*-[2-[[3-(4-bromophenyl)-2-propenyl]amino]ethyl]-5-isoquinoline sulfonamide dihydrochloride (H-89) (Sigma-Aldrich) was prepared in 0.9% sterile saline to a final concentration of 5 *μ*M for bilateral injection into the CA1 of the hippocampus (1 *μ*l/side).

Beginning 24 hours after microinjection of A*β*_1–42_ or ACF, BPN14770 was dosed by oral gavage for 14 days. H-89 was microinjected into the CA1 of the hippocampus 30 minutes before BPN14770 administration (as shown in the diagram above). The behavioral tests were performed 1 hour after the last treatment. Cognitive behavior did not differ between the BPN14770 group and the group that received BPN14770 plus microinjection of 0.9% sterile saline into the CA1 of the hippocampus when mice were subjected to chronic treatment of 14 days. This indicates that repeated hippocampal injection did not induce brain damage.

A total of 84 mice were randomly divided into seven groups (12 mice in each group) for behavioral tests. The groups were vehicle 1 (ACF) + vehicle 2 (distilled water); A*β*_1–42_ + vehicle 2; A*β*_1–42_ + BPN14770 (0.003 mg/kg); A*β*_1–42_ + BPN14770 (0.01 mg/kg); A*β*_1–42_ + BPN14770; A*β*_1–42_ + H-89; and A*β*_1–42_ + H-89 + BPN14770 (0.03 mg/kg). Each group of mice was divided randomly into two cohorts after the behavioral tests were completed. One cohort of mice (six mice in each group) was used for morphologic experiments (rapid Golgi staining), and the other cohort of mice (six mice in each group) was used for neurochemical and molecular biologic experiments.

#### Behavioral Testing.

Behavioral testing was conducted on days 19, 20, and 21 after implantation of the in-dwelling cannula. On day 19, the mice were gavaged with BPN14770 or vehicle, and then 1 hour later they were evaluated in the Y-maze test. The same mice were then evaluated in the acquisition trials of the Morris water maze (MWM) test and in the first probe trial 1 hour after the last block of acquisition trials. On day 20, the mice were gavaged with BPN14770 or vehicle and then 1 hour later were evaluated in the second probe trial 24 hours after the last block of acquisition trials. On day 21, mice were gavaged with BPN14770 or vehicle and 1 hour later tissues were harvested for morphologic and biomarker analysis.

#### Y-Maze Test.

Mice were placed at the intersection of the maze and allowed to explore the space freely for 5 minutes (Zhang et al., 2018). The arm entries were recorded automatically for a period of 5 minutes by an automatic image analytic system (Noldus Information Technology, Inc., Leesburg, VA). The maximum alternation was calculated as the total number of entries minus 2. The percentage of alternation was calculated as (actual alternations/maximum alternations) × 100 to assess working memory.

#### Morris Water Maze Test.

The maze was a circular plastic pool with a circular platform that was placed in one of the four quadrants. The pool was filled with opaque water (26 ± 1°C water mixed with milk powder) to 1 cm above the platform. Acquisition trials consisting of six blocks were provided to train the mice to escape to the hidden platform. Each block consisted of three trials and mice were given 60 seconds to locate the platform in each trial (Xu et al., 2015). Twenty minutes of resting time was granted to each mouse before moving on to the next block. A mouse was guided to the platform and allowed to sit on it for 15 seconds if it failed to find the platform during the specified duration. The probe test (with the platform removed) was performed 1 or 24 hours after the last acquisition trial to examine short- and long-term memory. Swimming speed, latency to the platform, entries into the target quadrant, and duration in the target quadrant were recorded during the probe test.

#### Rapid Golgi Staining in the Hippocampus.

The staining was performed according to the protocols of the FD Rapid GolgiStain Kit (FD NeuroTechnologies, Ellicott City, MD) and our previous studies (Paxinos and Franklin, 2004; Xu et al., 2015). After staining, brain samples containing the entire hippocampus (−1.4 to −2.4 mm from the bregma) were serially sectioned into 100 *μ*m coronal slices with a freezing microtome. Brain sections were dehydrated in alcohol, cleared in xylene, and mounted in neutral balsam. For tracing selected neurons for computerized image analysis, a camera lucida drawing tube attached to an Olympus microscope BX51 (Olympus, Tokyo, Japan) was used. The center of the soma served as the reference dot, while the total dendritic length and the number of dendrites were quantified every 50 *μ*m from the soma up to 400 *μ*m. The exact length of the dendritic segment divided by the number of spines along the length was calculated as the spine density, e.g., spines/10 *μ*m (Shankaranarayana Rao et al., 2001; Vyas et al., 2002).

#### Western Blot Analysis.

The hippocampal tissues were homogenized in radioimmunoprecipitation assay lysis buffer supplemented with protease and phosphatase inhibitors. Then, 45 *µ*g protein was resolved by 10% SDS-PAGE and transferred to polyvinylidene difluoride membranes. The blots were blocked with 5% bovine serum albumin for 1 hour before 4°C overnight incubation with appropriate primary antibodies. The blots then were washed with Tris-buffered saline and Tween-20 for three times before incubating with secondary antibodies for 1 hour at room temperature. Finally, chemiluminescence reagent (Thermo Fisher Scientific) was used for detection enhancement and visualization was achieved using Quantity One 1-D Analysis Software (Bio-Rad Laboratories, Inc., Hercules, CA).

#### Statistical Analysis.

The results are presented as mean ± S.E.M. and were analyzed by GraphPad Prism. Unless otherwise specified, data were analyzed by one-way ANOVA followed by a post hoc Dunnett’s test. The data from the acquisition trials in the MWM were analyzed by two-way ANOVA. A significance value of *P* < 0.05 was used for the statistical tests.

## Results

### 

#### BPN14770 Prevented A*β*-Induced Spatial Memory Impairment in the Y-Maze Spontaneous Alternation Test.

**Fig. 1.**
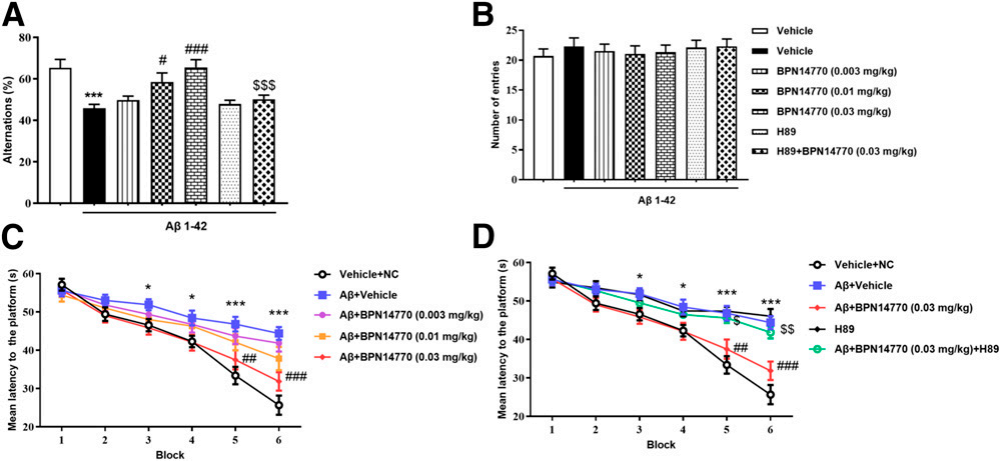
BPN14770 prevented A*β*-induced spatial memory impairment in the Y-maze test and training (acquisition) trials of the Morris water maze test. (A) Oligomeric A*β*_1–42_ decreased alternations in the Y-maze test, and this was reversed by BPN14770 in a dose-dependent manner. The effect of BPN14770 was blocked by pretreatment with the PKA inhibitor H-89. H-89 was administered each day 30 minutes before oral administration of BPN14770. (B) The number of entries, a measure of motor activity, was not changed by the drug treatments. Results are shown as mean ± S.E.M. (*n* = 12 per group). Results were analyzed by one-way ANOVA followed by a post hoc Dunnett’s test. Shown are the results for the alternations (*F*_6,77_ = 7.762, *P* < 0.001) and number of entries (*F*_6,77_ = 0.243, *P* = 0.96). ***P < 0.001 vs. vehicle-treated control group; ^#^*P* < 0.05, ^###^*P* < 0.001 vs. vehicle-treated A*β*_1–42_ group; ^$$$^*P* < 0.001 vs. BPN14770-treated A*β*_1–42_ group. (C and D) Learning curves in the Morris water maze test. Learning to locate the position of the hidden platform is impaired in mice treated with oligomeric A*β*_1–42_ and this is reversed by BPN14770 in a dose-dependent manner. Results are presented as mean ± S.E.M. (*n* = 12 per group). Results were analyzed by two-way ANOVA followed by a post hoc Dunnett’s test. (C) Shown are the results for factor treatment T (*F*_4,330_ = 15.78, *P* < 0.001), factor block B (*F*_5,330_ = 65.60, *P* < 0.001), and factor T × B (*F*_20,330_ = 2.346, *P* = 0.001). (D) Shown are the results for factor treatment T (*F*_4,330_ = 27.85, *P* < 0.001), factor block B (*F*_5,330_ = 72.89, *P* < 0.001), and factor T × B (*F*_20, 330_ = 4.058, *P* < 0.001). *P < 0.05, ***P < 0.001 vs. vehicle-treated control group; ^##^*P* < 0.01, ^###^*P* < 0.001 vs. vehicle-treated A*β*_1–42_ group; ^$^*P* < 0.05, ^$$^*P* < 0.01 vs. BPN14770-treated A*β*_1–42_ group.

The effect of BPN14770 on A*β*_1–42_-induced memory impairment was evaluated in the Y-maze spontaneous alternation test as shown in Fig. 1, A–C. Mice in the A*β*_1–42_ groups were implanted with in-dwelling cannula inserted into the CA1 region of the hippocampus, allowed to recover from surgery for 7 days, and then were administered bilateral injections of oligomeric A*β*_1–42_. Control mice were implanted with the in-dwelling cannula but received bilateral injections of ACF. Both groups of mice were gavaged daily with vehicle starting 1 day after receiving the hippocampal microinjections. Mice that received bilateral microinjections of oligomeric A*β*_1–42_ exhibited a lower percentage of alternations compared with mice that received bilateral microinjections of ACF (*P* < 0.001). Once daily oral treatment with BPN14770 starting on the day after intrahippocampal microinjection of oligomeric A*β*_1–42_ significantly attenuated the impairment of spontaneous alternation behavior induced by A*β*_1–42_ at doses of 0.01 and 0.03 mg/kg (*P* < 0.001). To assess the role of PKA, the mice were treated daily with the PKA inhibitor H-89 by microinjection through the in-dwelling cannula 30 minutes prior to oral administration of BPN14770. The memory enhancing effect of BPN14770 was blocked by pretreatment with the PKA inhibitor (*P* < 0.001), while bilateral microinjection of H-89 did not have a significant effect on the memory impairment induced by A*β*_1–42_. There was no significant difference in the number of arm entries across the groups, which indicated that BPN14770 did not affect motor activity (Fig. 1B).

#### BPN14770 Prevented A*β*-Induced Working Memory Impairment in the Morris Water Maze Test.

In the training blocks of the MWM test (acquisition sessions), mice treated with intrahippocampal microinjection of oligomeric A*β*_1–42_ were slower to reach the hidden platform in blocks 3, 4, 5, and 6 compared with the controls (*P* < 0.05 or *P* < 0.001) (Fig. 1C). Microinjection of oligomeric A*β*_1–42_ into the hippocampus did not impair motor activity since there was no difference in mean swimming velocity (Supplemental Fig. 1). Daily treatment with BPN14770 prevented the impairment of acquisition at a dose of 0.03 mg/kg, e.g., the latency to touch the platform for the BPN14770-treated (0.03 mg/kg) mice was significantly shorter than that of the vehicle-treated A*β*_1–42_ group from the fifth to sixth block (*P* < 0.01 or *P* < 0.001). The effects of BPN14770 on acquisition were blocked by pretreatment with H-89 (blocks 5 and 6; *P* < 0.05 or *P* < 0.01) (Fig. 1D).

**Fig. 2.**
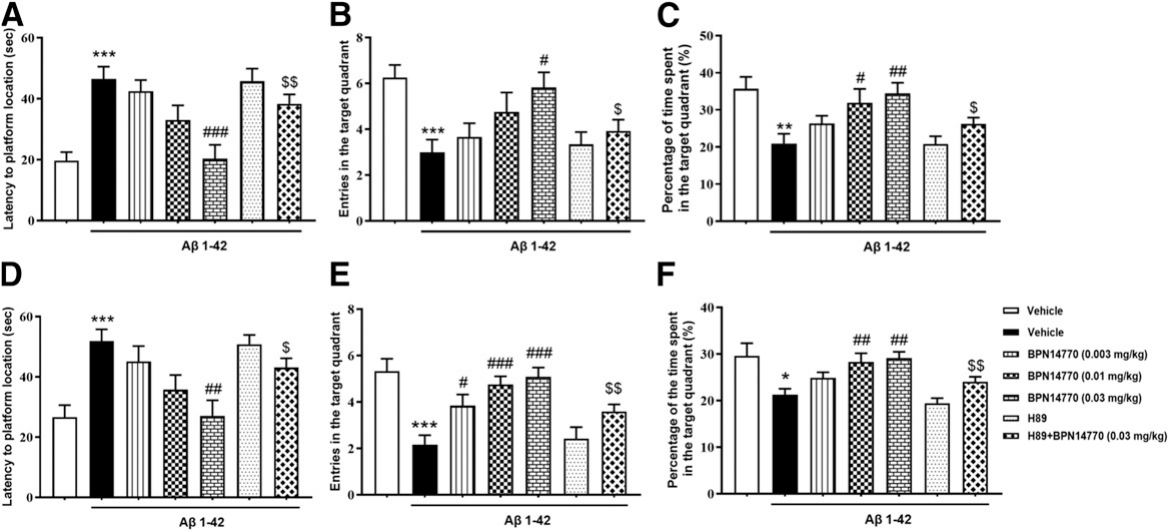
BPN14770 prevented A*β*-induced memory deficit 1 and 24 hours after the training session. (A–C) A*β*-treated mice showed impaired memory 1 hour after the training session and this was prevented by BPN14770. H-89 blocked the effects of BPN14770 at a dose of 0.03 mg/kg. Results are shown as mean ± S.E.M. (*n* = 12 per group). Results were analyzed by one-way ANOVA followed by a post hoc Dunnett’s test. Shown are the results for latency to platform (*F*_6,77_ = 8.375, *P* < 0.001), entries in the target quadrant (*F*_6,77_ = 4.161, *P* = 0.001), and percentage of time spent in the target quadrant (*F*_6,77_ = 5.070, *P* < 0.001). **P < 0.01, ***P < 0.001 vs. vehicle-treated control group; ^#^*P* < 0.05, ^##^*P* < 0.01, ^###^*P* < 0.001 vs. vehicle-treated A*β*_1–42_ group; ^$^*P* < 0.05, ^$$^*P* < 0.01 vs. BPN14770-treated A*β*_1–42_ group. (D–F) A*β*-treated mice showed memory deficits 24 hours after the training session and this was prevented by BPN14770. H-89 blocked the effects of BPN14770 at a dose of 0.03 mg/kg. Results are shown as mean ± S.E.M. (*n* = 12 per group). Results were analyzed by one-way ANOVA followed by a post hoc Dunnett’s test. Shown are the results for latency to platform (*F*_6,77_ = 6.054, *P* < 0.001), entries in the target quadrant (*F*_6,77_ = 8.443, *P* < 0.001), and percentage of time spent in the target quadrant (*F*_6,77_ = 5.972, *P* < 0.001). *P < 0.05, ***P < 0.001 vs. vehicle-treated control group; ^#^*P* < 0.05, ^##^*P* < 0.01, ^##^*P* < 0.001 vs. vehicle-treated A*β*_1–42_ group; ^$^*P* < 0.05, ^$$^*P* < 0.01 vs. BPN14770-treated A*β*_1–42_ group.

To test short-term memory retention, the animals were tested 1 hour after the last acquisition trial in a probe trial in which the hidden platform was removed. The mice treated with oligomeric A*β*_1–42_ and gavaged with vehicle took significantly longer to swim to the previous platform location and made fewer crossings in the target quadrant than control mice that received microinjections of ACF (*P* < 0.001; *P* < 0.001) (Fig. 2, A and B). The percentage of the time spent in the target quadrant also was significantly lower in A*β*_1–42_-treated mice than mice microinjected with ACF (*P* < 0.01) (Fig. 2C). Once daily oral gavage with BPN14770 dose dependently reduced the A*β*_1–42_-induced memory loss as evidenced by a decrease in the latency to the platform location (*P* < 0.001), an increase in the entries into the target quadrant (*F*_3,44_ = 3.426, *P* < 0.05), and an increase in the time spent in the target quadrant (*P* = 0.01). Pretreatment with H-89 once again blocked the protective effects of BPN14770 on memory loss (*P* < 0.01, *P* < 0.05, and *P* < 0.05, respectively); a second probe trial test for long-term memory that was performed 24 hours after the training session yielded similar results as shown in Fig. 2, D–F. BPN14770 at doses of 0.003, 0.01, and 0.03 mg/kg reversed impairment of spatial memory consolidation and retrieval induced by A*β*_1–42_ as shown by a shorter time to reach the previous platform location (*P* < 0.01) (Fig. 2D), more crossings (*F*_3,44_ = 9.953, *P* < 0.001) (Fig. 2E), and longer time spent in the target quadrant (*P* < 0.01) (Fig. 2F). The benefit of BPN14770 was blocked by H-89 (*P* < 0.05, *P* < 0.01, and *P* < 0.01, respectively). Notably, there was no significant difference in swimming speed across groups in the training session (*F*_6,77_ = 0.430, *P* = 0.857) or 1-hour (*F*_6,77_ = 0.526, *P* = 0.785) and 24-hour (*F*_6,77_ = 0.411, *P* = 0.870) probe trails (Supplemental Fig. 1, A–C). The total swimming distance they traveled also did not show any significant difference in the 1-hour (*F*_6,77_ = 0.526, *P* = 0.785) or 24-hour (*F*_6,77_ = 0.411, *P* = 0.870) probe trails (Supplemental Fig. 1, D and E). This indicates that there was no impairment of vision or motor activity across the treatments.

#### BPN14770 Ameliorated A*β*_1–42_-Induced Neuronal Atrophy in the CA1 of the Hippocampus.

**Fig. 3.**
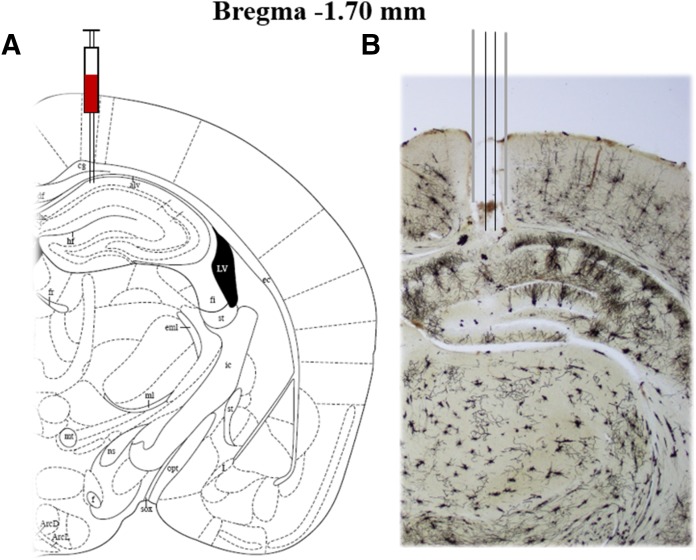
Photomicrographs of representative cannula placements in the hippocampus. (A) Sections are according to the atlas (Paxinos and Franklin, 2004); (B) Rapid Golgi staining in the hippocampus section showing the cannula track.

**Fig. 4.**
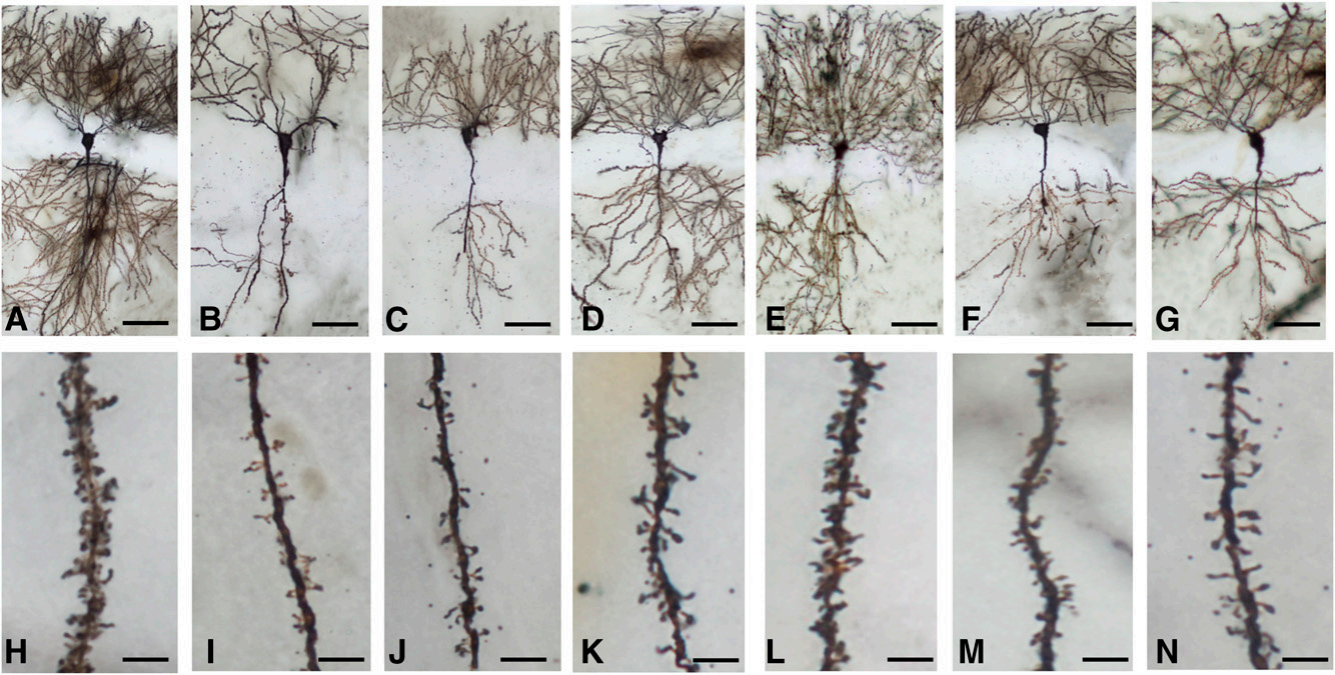
Photomicrographs of representative Golgi-impregnated hippocampal CA1 pyramidal neurons [(A–G), scale bar, 25 *μ*m] and the apical dendrite [(H–N), scale bar, 5 *μ*m] from each of the treatment groups. (A and H) Vehicle 1 (ACF) + vehicle 2 (distilled water); (B and I) A*β*_1–42_ + vehicle 2; (C and J) A*β*_1–42_ + BPN14770 (0.003 mg/kg); (D and K) A*β*_1–42_ + BPN14770 (0.01 mg/kg); (E and L) A*β*_1–42_ + BPN14770 (0.03 mg/kg); (F and M) A*β*_1–42_ + H-89; and (G and N) A*β*_1–42_ + H-89 + BPN14770 (0.03 mg/kg).

**Fig. 5.**
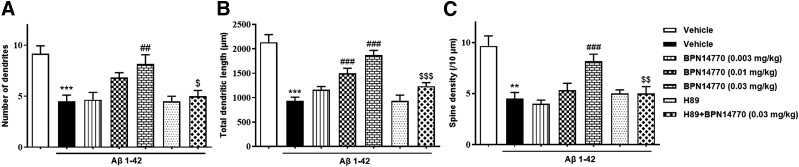
BPN14770 prevented A*β*-induced neuronal atrophy in the hippocampal CA1 pyramidal neurons. (A) Number of dendrites; (B) total dendritic length and (C) spine density (per 10 *μ*m distances) were measured. Results are expressed as mean ± S.E.M. (*n* = 6 per group). Results were analyzed by one-way ANOVA followed by a post hoc Dunnett’s test. Shown are the results for the number of dendrites (*F*_6,35_ = 8.339, *P* < 0.001), total dendritic length (*F*_6,35_ = 19.49, *P* < 0.001), and spine density (*F*_6,35_ = 10.31, *P* < 0.001). **P < 0.01, ***P < 0.001 vs. vehicle-treated control group; ^##^*P* < 0.05, ^###^*P* < 0.001 vs. vehicle-treated A*β*_1–42_ group; ^$^*P* < 0.05, ^$$^*P* < 0.01, ^$$$^*P* < 0.001 vs. BPN14770-treated A*β*_1–42_ group.

The morphology of CA1 pyramidal neurons in the hippocampus was severely affected by exposure to oligomeric A*β*_1–42 _(Fig. 3). This can be seen by the detailed segmental analysis of the number of dendritic branch points, total dendritic length, and dendritic spine density as a function of radial distance from the cell soma (150–350 *µ*m) (). This quantitative analysis found significant differences across all three parameters between mice treated with oligomeric A*β*_1–42_ compared with mice given bilateral microinjections of ACF (*P* < 0.001, *P* < 0.001, and *P* < 0.01) (Figs. 4 and 5). BPN14770 prevented oligomeric A*β*_1–42_ toxicity in a dose-dependent manner. BPN14770 at doses of 0.003, 0.01, and 0.03 mg/kg significantly increased the total number of dendrites (*P* < 0.01), dendritic length (*P* < 0.001), and spine density (*P* < 0.01). Daily administration of the PKA inhibitor H-89 prior to administration of BPN14770 blocked the protective effect of BPN14770. The improvement due to treatment with BPN14770 was positively correlated with the total number of dendrites and dendritic length (Supplemental Figs. 2–4), but not the spine density of CA1 neurons (Supplemental Fig. 4C).

#### BPN14770 Prevented A*β*_1–42_-Induced Decreases in Plasticity-Related Protein Expression in the Hippocampus.

**Fig. 6.**
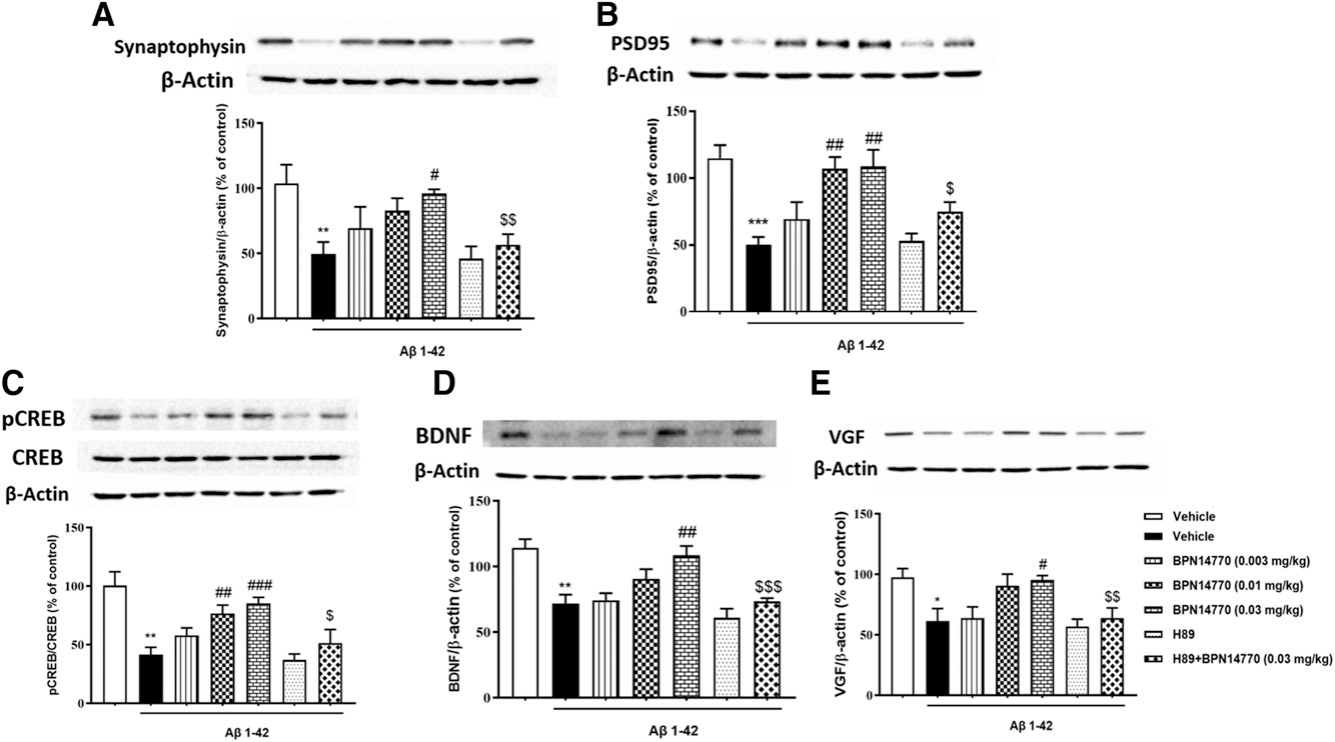
Immuno-blot analyses for synaptophysin (A), PSD-95 (B), pCREB/CREB (C), BDNF (D), and VGF (E) expression in the hippocampus. Results are expressed as mean ± S.E.M. (*n* = 6 per group). Results were analyzed by one-way ANOVA followed by a post hoc Dunnett’s test. Shown are the results for synaptophysin (*F*_6,35_ = 4.514, *P* = 0.002), PSD-95 (*F*_6,35_ = 8.739, *P* < 0.001), pCREB/CREB (*F*_6,35_ = 8.621, *P* < 0.001), BDNF (*F*_6,35_ = 10.10, *P* < 0.001), and VGF (*F*_6,35_ = 4.845, *P* = 0.001). *P < 0.05, **P < 0.01, ***P < 0.001 vs. vehicle-treated control group; ^#^*P* < 0.05, ^##^*P* < 0.01, ^###^*P* < 0.001 vs. vehicle-treated A*β*_1–42_ group; ^$^*P* < 0.05, ^$$^*P* < 0.01, ^$$$^*P* < 0.01 vs. BPN14770-treated A*β*_1–42_ group.

Bilateral injection of oligomeric A*β*_1–42_ reduced two biomarkers of synaptic density, synaptophysin and postsynaptic density protein 95 (PSD-95). As shown in Fig. 6, A and B, synaptophysin and PSD-95 were significantly reduced in mice treated with oligomeric A*β*_1–42_ compared with mice that received microinjection of ACF (*P* < 0.01 or *P* < 0.001). Daily dosing with BPN14770 prevented the loss of synaptophysin and PSD-95 in a dose-dependent manner (*P* < 0.05 for synaptophysin; *P* = 0.001 for PSD-95). Levels of synaptophysin and PSD-95 were positively correlated with the improvement in memory (Supplemental Figs. 5–7).

Daily administration of H-89 prior to dosing with BPN14770 blocked the protective effect of BPN14770 (*P* < 0.01 or *P* < 0.05). Thus, the protective effect of BPN14770 on biomarkers of synaptic density is consistent with the protective effect of the compound on dendritic morphology.

#### BPN14770 Prevented A*β*-Induced Decreases in Phosphorylated CREB/CREB, BDNF, and Nerve Growth Factor Inducible Protein Expression in the Hippocampus.

Previous studies have shown that BPN14770 increases signaling through cAMP, which leads to activation of PKA and phosphorylation of CREB (Zhang et al., 2018). Bilateral microinjection of oligomeric A*β*_1–42_ into the hippocampus impairs signaling through this pathway. As shown in Fig. 6C, oligomeric A*β*_1–42_ significantly reduced the ratio of phosphorylated CREB (pCREB)/CREB (*P* < 0.01) compared with microinjection of ACF, and this was prevented by treatment with BPN14770 in a dose-dependent manner (*P* < 0.001). Moreover, the effect of BPN14770 on CREB phosphorylation was blocked by pretreatment with the PKA inhibitor H-89 (*P* < 0.05). Bilateral microinjection of oligomeric A*β*_1–42_ also decreases expression of BDNF and nerve growth factor inducible protein (VGF), which are downstream effector molecules of cAMP signaling through CREB phosphorylation (*P* < 0.01; *P* < 0.05) (Fig. 6, D and E). Daily dosing with BPN14770 dose dependently prevented the loss of BDNF and VGF in the hippocampus at 0.03 mg/kg (*P* < 0.01; *P* < 0.05). The increase in pCREB/CREB, BDNF, and VGF expression in the hippocampus was positively correlated with the improvement in memory (Supplemental Figs. 5–7). The effects of BPN14770 on BDNF and VGF were blocked by pretreatment with the PKA inhibitor H-89 (*P* < 0.001; *P* < 0.01).

## Discussion

This study demonstrates that a phosphodiesterase-4D allosteric inhibitor protects against memory loss and neuronal atrophy induced by microinjection of oligomeric A*β*_1–42_ into the hippocampus of hPDE4D mice. The use of hPDE4D mice allowed us to explore PDE4D pharmacology at doses of BPN14770 that do not appreciably inhibit PDE4 subtypes A and B, the other subtypes of PDE4 present in the brain (Zhang et al., 2018; Gurney et al., 2019). Very low doses BPN14770 (0.01 and 0.03 mg/kg) prevented the impairment of memory acquisition and retrieval in the MWM and Y-maze tests caused by microinjection of oligomeric A*β*_1–42_. The morphologic studies showed that BPN14770 protected hippocampal neurons against oligomeric A*β*_1–42_ neurotoxicity, as evidenced by preservation of the number of dendrites, total dendritic length, and spine density in the CA1 of the hippocampus. BPN14770 also prevented A*β*-induced reduction of pCREB/CREB, BDNF, and VGF and deficits in synaptic marker proteins such as synaptophysin and PSD-95 in the hippocampus. The protective effect of BPN14770 was prevented by pretreatment with the PKA inhibitor H-89, which suggests that the benefit of BPN14770 is mediated through PKA activation.

The Morris water maze is an aversively motivated spatial learning and memory paradigm that has been used extensively to study the neurobiology of cognitive performance in rodents. The learning and memory paradigms in this task are dependent on two major aspects of functional processing: motivation (reward related and climbing onto the platform to escape from the water) and information processing (acquisition, consolidation, and retrieval of the platform location) (Lubbers et al., 2007; Zhang et al., 2013). In the present study, mice treated with oligomeric A*β*_1–42_ were slower to reach the platform in the acquisition phase, and in the probe trials they spent less time in the target quadrant where the platform previously was located. BPN14770 dose dependently prevented A*β*_1–42_-induced memory loss in hPDE4D mice, as shown by a progressive decrease in latency to platform in the training session (acquisition trial) and probe trials, and by an increase in exploration time in the target quadrant in the probe trial, although it is difficult to reflect aspects of motivation and perseverance in this task. A protective effect of BPN14770 also was observed in the Y-maze test. Our present findings are corroborated by our previous report that BPN14770 increases brain cAMP levels and improves memory function through a cAMP- and PKA-dependent pathway (Zhang et al., 2018). This new study also is consistent with our earlier finding that knockdown of long-form PDE4D in the cortex significantly improves cognitive function in stressed animals (Wang et al., 2013). This further supports the idea that PDE4D is a key modulator of memory processes and that a selective PDE4D allosteric inhibitor prevents oligomeric A*β*_1–42_ neurotoxicity and improves memory function by stimulating compensatory synaptic mechanisms.

Our study shows that microinjection of oligomeric A*β*_1–42_ into the CA1 of the hippocampus induces dendritic abnormalities with associated impairment of memory function. This supports the idea that alterations in hippocampal circuitry are critical for understanding disorders involving memory impairment and dementia (Sapolsky et al., 1986). The hippocampus is susceptible to abnormal aging processes such as extracellular A*β* deposition, and thus exhibits a crucial role in regulating memory functions, including the formation of stable declarative (or explicit) memory in humans and spatial (or relational/contextual) memory in rodents (Xu et al., 2009). Recent evidence suggests that synaptic loss in the hippocampus is strongly associated with cognitive dysfunction (Scheff and Price, 2003, 2006). Thus, therapeutic treatments that improve synaptic plasticity may have potential therapeutic benefit for patients with early or prodromal Alzheimer’s disease. In the present study, microinjection of oligomeric A*β*_1–42_ into the hippocampus resulted in serious neuronal atrophy. This was prevented by daily treatment with BPN14770 as shown by the dose-dependent protection of the number of dendrites, total dendritic length, and spine density. Although we did not find a relationship between memory improvement and spine density, this did not affect the correlation between memory behavior and plasticity changes in mature neurons in our further study. The main reason may be that the development of neurons is a highly complex process and not all of the synapses would become mature neurons. The further correlation analysis between the behavioral phenotype and dendritic morphology in the mature hippocampal neurons indicates that the benefit of BPN14770 for memory improvement was positively correlated to the structure and function of CA1 neurons. These effects of BPN14770 corroborate our previous observations, which suggested that BPN14770 augments long-term potentiation in the hippocampus, one of the initial and transient changes in synaptic plasticity that underlies early stages of memory deficits associated with Alzheimer’s disease (Wang et al., 2004; Zhang et al., 2018). Previously, BPN14770 was shown to ameliorate behavioral phenotypes in a mouse model of fragile X syndrome, while also improving maturation of dendritic spine morphology on layer II/III pyramidal cells in the dorsolateral prefrontal cortex (Gurney et al., 2017). Knockdown of PDE4D mRNA also stimulates synapse maturation on layer II/III pyramidal cells in the dorsolateral prefrontal cortex (Baumgärtel et al., 2018).

The effect of BPN14770 on the preservation of dendritic morphology was corroborated by the preservation of pre- and postsynaptic proteins, e.g., synaptophysin and PSD-95, which are markers of synaptic density. The preservation of these two synapse-related proteins suggests that the effects of BPN14770 on neuroplasticity not only apply to structural remodeling, but also to the functional plasticity of the brain. Moreover, this effect of BPN14770 on A*β*-induced neuronal atrophy was prevented by pretreatment with the PKA inhibitor H-89, which further supports the critical role of PKA-mediated cell signaling in neuroplasticity. These results agree with previous studies, which demonstrated that activation of the cAMP/PKA/CREB pathway results in facilitation of synaptic plasticity and memory formation (Gong et al., 2004; Zhang et al., 2018). CREB phosphorylation upregulates synaptic plasticity-related proteins, such as synaptophysin and PSD-95; therefore, it supports long-lasting alterations in synaptic connectivity and memory formation (Prickaerts et al., 2002). Our findings suggest that the reduction in PKA-pCREB signaling induced by oligomeric A*β*_1–42_ is reversed by treatment with BPN14770, which in turn promotes enhancement of memory and neuronal remodeling.

The BDNF gene contains a cAMP response element to which phosphorylated CREB binds, thereby enhancing transcription (Xu et al., 2006). Clinical observations suggest that phosphorylated CREB is aberrantly sequestered in hippocampal neurons in Alzheimer’s disease with generalized disruption of CREB-mediated signaling (Satoh et al., 2009), BDNF regulation of synaptic plasticity, and neurogenesis (Cunha et al., 2010; Hollands et al., 2016; Moreno-Jiménez et al., 2019). VGF is a BDNF-inducible neuropeptide that plays an important role in hippocampal neurogenesis and synaptic plasticity (Alder et al., 2003; Bozdagi et al., 2008). A significant decrease in VGF level has been observed in the cerebrospinal fluid, prefrontal cortex, and hippocampus of patients with Alzheimer’s disease (Rüetschi et al., 2005; Thakker-Varia et al., 2010; Ramos et al., 2014). Our results indicate that oligomeric A*β*_1–42_ significantly decreases BDNF and VGF expression in the hippocampus, while BPN14770 prevents such deficits.

In summary, modeling of oligomeric A*β*_1–42_ neurotoxicity in mice indicates that BPN14770 triggers multiple compensatory mechanisms that reduce impairment of memory, damage to dendritic morphology, deficits in synaptic proteins, impaired signaling through CREB phosphorylation, and production of neurotrophic signaling molecules such as BDNF and VGF.

## Abbreviations

A*β*: amyloid beta
ACF: artificial cerebrospinal fluid
BDNF: brain-derived neurotrophic factor BPN14770 2-(4-((2-(3-Chlorophenyl)-6-(trifluoromethyl)pyridin-4-yl)methyl)phenyl)acetic Acid
CREB: cAMP-response element binding protein
hPDE4D: humanized phosphodiesterase-4D
H-89: *N*-[2-(p-Bromocinnamylamino)ethyl]-5-isoquinolinesulfonamide dihydrochloride
MWM: Morris water maze
pCREB: phosphorylated cAMP-response element binding protein
PDE4: phosphodiesterase-4
PDE4D: phosphodiesterase-4D
PKA: protein kinase A
PSD-95: postsynaptic density protein 95
VGF: nerve growth factor inducible protein
